# Optimizing preventive interventions in child drowning based on caregivers' lived experiences

**Published:** 2023-12

**Authors:** Faeze Kobrai-Abkenar, Parand Pourghane

**Affiliations:** ^*a* Department of Nursing, Zeynab (P.B.U.H) School of Nursing and Midwifery, Guilan University of Medical Sciences, Rasht, Iran. ^

## Abstract

**Background:**

In developed and developing societies, accident in children is considered an important issue of public health and global development because they are one of the most vulnerable groups involved in accidents. Falling into the water and the subsequent drowning is an unintentional accident that can be prevented. The present study aims to analyze the results of child caregivers' experiences of drowning in a review of qualitative research.

**Methods:**

The method applied in the present study was a narrative review. Initially, a variety of different sources were employed to find the related literature. The sources included Science Direct, PubMed, Scopus, Google Scholar, SID, Irandoc, Magiran, and Iranmedex with keywords combination of “Child drowning”, “Parents”, “Perception”, “Drowning”, “Prevention”, “Qualitative study”, and “Experiences“. The inclusion criteria were publication time between 2000 and 2023, being relevant to the purpose of the study, being in English or Farsi language, and being available in full text. The texts were examined by two researchers independently. Finally, among the 45 articles, 12 articles were selected and analyzed.

**Results:**

After reviewing 12 articles, the lived experiences of caregivers to prevent the drowning of children were classified in response to five basic questions: “Who drowns?” (Who?), “Where do children drown?” (Where), “When do children drown?” (When?), “Why do children drown?” (Why?), “What are the ways to prevent drowning?” (What?).

**Conclusions:**

The present study shows that identifying the high-risk groups among children, high-risk places and times, and the causes of children's drowning, provide the information needed to determine goals, plans, and design preventive interventions in various societies. In developed as well as in developing countries, these interventions can be classified at three levels: personal, community, and NGOs, and the government in order to provide financial, human, and physical resources.

**Keywords:**

Experience, Drowning, Child, Article review, Qualitative research

